# The miRNA Content of Exosomes Released from the Glioma Microenvironment Can Affect Malignant Progression

**DOI:** 10.3390/biomedicines8120564

**Published:** 2020-12-03

**Authors:** Federica Caponnetto, Emiliano Dalla, Damiano Mangoni, Silvano Piazza, Slobodanka Radovic, Tamara Ius, Miran Skrap, Carla Di Loreto, Antonio Paolo Beltrami, Ivana Manini, Daniela Cesselli

**Affiliations:** 1 Department of Medicine, University of Udine, 33100 Udine, Italy; emiliano.dalla@uniud.it (E.D.); carla.diloreto@uniud.it (C.D.L.); antonio.beltrami@uniud.it (A.P.B.); daniela.cesselli@uniud.it (D.C.); 2 Central RNA Laboratory, Istituto Italiano di Tecnologia (IIT), 16163 Genova, Italy; damiano.mangoni@iit.it; 3 International Center for Genetic Engineering and Biotechnology (ICGEB), 34149 Trieste, Italy; piazza@icgeb.org; 4 IGA Technology Services, S.R.L., 33100 Udine, Italy; sradovic@igatechnology.com; 5 Neurosurgery Unit, Department of Neurosciences, University Hospital of Udine, 33100 Udine, Italy; tamara.ius@asufc.sanita.fvg.it (T.I.); skrap@asufc.sanita.fvg.it (M.S.); 6 Institute of Pathology, University Hospital of Udine, 33100 Udine, Italy

**Keywords:** exosomes, tumor microenvironment, microRNA, low-grade gliomas, stratification criteria

## Abstract

Low-grade gliomas (LGG) are infiltrative primary brain tumors that in 70% of the cases undergo anaplastic transformation, deeply affecting prognosis. However, the timing of progression is heterogeneous. Recently, the tumor microenvironment (TME) has gained much attention either as prognostic factor or therapeutic target. Through the release of extracellular vesicles, the TME contributes to tumor progression by transferring bioactive molecules such as microRNA. The aim of the study was to take advantage of glioma-associated stem cells (GASC), an in vitro model of the glioma microenvironment endowed with a prognostic significance, and their released exosomes, to investigate the possible role of exosome miRNAs in favoring the anaplastic transformation of LGG. Therefore, by deep sequencing, we analyzed and compared the miRNA profile of GASC and exosomes obtained from LGG patients characterized by different prognosis. Results showed that exosomes presented a different signature, when compared to their cellular counterpart and that, although sharing several miRNAs, exosomes of patients with a bad prognosis, selectively expressed some miRNAs possibly responsible for the more aggressive phenotype. These findings get insights into the value of TME and exosomes as potential biomarkers for precision medicine approaches aimed at improving LGG prognostic stratification and therapeutic strategies.

## 1. Introduction

Adult diffuse low-grade gliomas (LGG) are classified and graded based on histological and molecular parameters according to the World Health Organization (WHO) classification of brain tumors [1]. Although glioblastoma (WHO grade IV) remains the most aggressive and deadliest tumor of the central nervous system, with a median survival time of 12.2–18.2 months [2], WHO grade II gliomas also represent a clinical challenge, due to their highly variable behavior [3,4]. Indeed, some LGG remain indolent for years, while others rapidly progress to glioblastoma, therefore, the survival of LGG patients ranges from 15 years to 1 year [3,4]. Comprehensive genome studies have contributed to a better prognostic stratification of lower grade glioma [5,6,7,8]. Specifically, diffuse gliomas isocitrate dehydrogenase (IDH) wildtype are characterized by the worst prognosis, oligodendrogliomas, IDH mutant, and 1p/19q co-deleted, by the best while diffuse astrocytoma IDH mutant by an intermediate prognosis [5,6,7,8]. However, even inside the same molecular group, there are differences in both risk and timing of malignant transformation. This makes it difficult to choose the therapeutic strategy, aimed at aggressively treating patients with a potentially more malignant neoplasm and avoiding adjuvant therapies to patients who could only progress after many years [3,4,9]. In fact, chemotherapy and especially radiotherapy, the only available adjuvant treatments, are burdened by severe side effects, such as the risk of late cognitive defects following radiotherapy. To refine the therapeutic approach towards personalized medicine, it is increasingly important to understand the molecular mechanisms driving the malignant evolution of LGG. To this aim, Bai et at. compared the genomic landscape of 41 WHO grade II IDH mutant astrocytomas to their higher-grade, progressed counterpart. Integrated genomic analyses, including whole-exome sequencing and copy number, gene expression, and DNA methylation profiling, showed, in the progressed sample, the activation of the MYC and RTK-RAS-PI3K pathways and upregulation of genes involved in the cell cycle transition, such as FOXM1 and E2F2, and the epigenetic silencing of genes related to Polycomb repressive complex 2 [10].

However, increasing evidence shows that cancer progression is driven, not only by genetic alterations, but also by the crosstalk established by tumor cells and the surrounding microenvironment [11,12,13,14].

In gliomas, the tumor microenvironment (TME) consists of non-tumor cells, such as endothelial cells, tumor-associated macrophages (TAMs), microglia, resident astrocytes, extracellular matrix components, proteins, and secreted molecules, all acting in the intercellular communication with tumor cells, thus modulating disease progression [15,16,17].

Important players of the potent cross talk between cancer cells and the TME are exosomes, small extracellular vesicles, with a diameter ranging between 50 to 150 nm [18,19,20]. They originate in the multivesicular body compartment of the endoplasmic reticulum and are released into the extracellular space and in the body fluids from many cell types [21]. After release, exosomes are internalized by neighboring or distant cells delivering their content in form of biologically active molecules (e.g., proteins, mRNAs, miRNAs, and lncRNAs), thus modifying the phenotype of target cells [22,23]. Indeed, there are increasing evidences that tumor cells release exosomes to create an environment supporting angiogenesis, invasion, tumor proliferation, and the premetastatic niche [24,25,26]. Moreover, it has also been shown that tumor-associated fibroblasts, through the release of exosomes, could induce in breast tumor cells the acquisition of a metastatic phenotype [27].

In glioblastoma, exosomes released by tumor cells, containing mRNA, miRNA, and angiogenic proteins, are able to act on endothelial cells, favoring the development of a tumor-permissive microenvironment [28]. Exosome miRNAs were associated with gliomagenesis by modulation of several signaling pathways [29,30,31]. miRNAs are non-coding single stranded RNAs of 18–27 nucleotides that influence various cellular processes decreasing the level of translation of their target mRNAs [32].

The packaging of miRNAs and proteins inside the exosomes is a selective process, as their content can be modulated at different stages of cancer development, mirroring the physiological state of the cell of origin, thus representing prognostic/predictive biomarkers [33,34,35].

Altogether, this knowledge contributes to increased attention on the TME as a novel source of therapeutic targets and markers of disease progression [36,37].

Cells residing in the TME would be less prone to develop drug-resistance, being devoid of mutations unlike cancer cells. Indeed, multiple TME-directed therapies are now under evaluation in clinical trials [38,39,40].

To deepen the comprehension of the role played by the TME on the malignant progression of LGG, we have taken advantage of our previously established in vitro model of glioma microenvironment, represented by glioma-associated stem cells and their released exosomes [41].

Glioma-associated stem cells (GASC), isolated both from human low- and high-grade glioma samples, are characterized by an undifferentiated mesenchymal phenotype, clonogenicity, and multipotency [41,42,43,44]. This cell population does not show genetic aberrations of the tumor of origin, and it is devoid of tumor-initiating properties, in vivo; nevertheless, GASC are characterized by the ability to grow in an anchorage-independent way, in vitro. Moreover, GASC from LGG were endowed with prognostic potential when comparing low-grade and high-grade gliomas’ specific surface markers. We also demonstrated that GASC could prognostically stratify LGG based on the expression of the NF-κB-related transcriptional program exerting their tumor-supporting properties through the stimulation of cytokines such as TNFα, IL1β, and IL-6, constituting new targets for novel adjuvant therapies.

In our in vitro model, GASC, from both HGG and LGG, were able to increase the in vitro aggressiveness of glioblastoma, through the release of exosomes, although those from LGG at a significant lower extent [41]. These data suggest that the degree of the tumor-supporting ability was proportional to the grade of malignancy of the tumor of origin. We have also identified a pro-migratory protein (SEMA-7A) able to promote the motility of glioma stem cells (GSC) in vitro, exposed on the surface of HGG-GASC exosomes [44].

As we have already demonstrated the prognostic value of GASC and assessed the importance of exosomes in sustaining the bidirectional crosstalk with tumor cells through the release of bioactive molecules, in this paper, we aimed at dissecting the miRNA content of exosomes derived from either GASC isolated from LGG characterized by a good prognosis or GASC isolated from LGG characterized by a rapid anaplastic transformation, regardless of WHO classification status. Taking advantage of deep sequencing and bioinformatic tools, we identified putative miRNAs possibly responsible for the heterogeneity in prognosis that characterizes LGG.

## 2. Experimental Section

The project had approval of the local ethics committee (Consents 102/2011/Sperand 196/2014/Em), and all experiments were conducted after written informed consent from all the patients enrolled according to the Declaration of Helsinki.

### 2.1. GASC Cell Culture, Supernatant Collection, and Exosomes Isolation

Human glioma samples derived from patients who underwent anaplastic transformation either within 48 months (LGG_BAD; *n* = 3) or ≥7 years (LGG_GOOD; *n* = 3) after surgery were collected at the Department of Neurosurgery, Santa Maria della Misericordia, University Hospital in Udine. GASC cells were isolated and cultured as in [41]. Briefly, glioma tissues were mechanically and enzymatically dissociated followed by a biophysical selection of cells smaller than 40 µm in diameter. Cells were expanded in vitro for three passages in selective expansion medium. For supernatant collection, cells were seeded at 6000 cells/cm^2^ in 100 mm Petri dishes in a 5% CO_2_ and 5% O_2_ incubator and maintained until 70–80% confluence (approximately 48 h) in exosome-depleted expansion medium.

Exosomes were isolated from the collected GASC supernatants using ExoQuick-TC Exosome precipitation solution (System Biosciences, Palo Alto, CA, USA), according to manufacturer’s instructions. Briefly, supernatants were collected after conditioning and centrifuged at 3000 *g* for 30 min at 4 °C to remove cells and debris; samples were then filtered through a 0.2 μm filter with a syringe to remove particles larger than 200 nm in diameter. Finally, supernatants were concentrated using centrifugal filter units (Amicon-Ultra15, Merck Millipore, Burlington, MA, USA) in regenerated cellulose membranes, with a pore size of 100 kDa, by centrifugation at 4700 *g* for 20 min at 4 °C. The ultra-filtered supernatants were then incubated with ExoQuick-TC Exosome precipitation solution (System Biosciences, Palo Alto, CA, USA) in a 5:1 dilution overnight at 4 °C and centrifuged at 1500 *g* for 15 minutes at 4 °C. The exosome pellets were resuspended in phosphate-buffered saline (PBS), milliQ water or radio immunoprecipitation assay (RIPA) buffer (NaCl 150 mM, 25 mM Tris-HCl, pH 7.6, 1% IGEPAL, (Octylphenoxypoly(ethyleneoxy)ethanol), 0.1% SDS, 1% SodiumDeoxycholate, 1 mM dithiothreitol, 1 mM Na2 VO4, 1 mM Sodium Fluoride, 0.5 mM Phenylmethanesulfonyl fluoride, Protease Inhibitor Cocktail, all from Sigma-Aldrich, Milan, Italy)for further analysis.

### 2.2. Nanoparticle Tracking Analysis

Size and particles’ concentration of exosome preparations were assessed using Nanosight (LM10, Malvern System Ltd., London, UK), equipped with a 405 nm laser. Analyses were performed as in [44]. Briefly, after optimizing camera level (15) and detection threshold (6), 1:1000 diluted exosome-enriched fractions were recorded for 60 s and analyzed by Nanoparticle Tracking Analysis (NTA) software (LM10, Malvern System Ltd., UK).

### 2.3. MACSPlex Analysis

Multiplex surface marker analysis was performed using MACSPlex Exosome, human, Kit (Miltenyi Biotec, Bergisch-Gladbach, Germany) according to manufacturers’ protocol. The short protocol for 1.5 mL reagent tubes was performed, using a combination of Allophycocyanin (APC)-stained CD9-, CD63-, and CD81-detection antibodies, which, in combination with a detection reagent, allows qualitative and semiquantitative analysis of 37 exosome surface epitopes. The analysis was performed in triplicate comparing the samples with the relative buffer (PBS) and the kit buffer, standardizing analysis for particles’ concentration (5 × 10^9^ particles). Flow cytometric analysis was performed on FASCanto (BD Biosciences, San Jose, CA, USA, http://www.bdbiosciences.com) and analyzed with the FACSDiva software (BD Biosciences, San Jose, CA, USA). For analysis, the median APC intensity (MFI) of all the 37 captured markers was normalized for PBS, buffer control, and isotypic control, and negative values were excluded.

### 2.4. Western Blot

Exosomes and cells proteins were obtained by lysis in RIPA buffer. Thirty micrograms of proteins were resolved on SDS-PAGE (Gel Tris-Glycine 10%), transferred, and immobilized on a 0.45 μm nitrocellulose membrane (Amersham, London, UK). Membranes were blocked by incubation for one hour at RT with 5% bovine serum albumin in Tris-Buffered Saline (TBS) (Tris-HCl 50mM, pH 7.4, NaCl 150 mM, all from Sigma-Aldrich, Italy) containing 0.2% Tween 20 (Sigma-Aldrich, Milan, Italy) and hybridized overnight at 4 °C with mouse monoclonal to Tumor Susceptibility Gene 101 protein (TSG101) (1:500 dilution) (Abcam, Cambridge, UK), rabbit monoclonal to calnexin (1:1000 dilution) (Cell Signaling Technology, Danvers, MA, USA).Primary antibodies were detected using horseradish peroxidase-linked secondary antibodies (anti-mouse 1:1000 dilution; anti-rabbit 1:5000 dilution) (DAKO, Cambridge, UK) and visualized using the enhanced chemiluminescent detection system (SuperSignal West Dura Extended Duration Substrate, Thermo Scientific, Walthman, MA, USA).

### 2.5. miRNA Extraction, Library Preparation, and Sequencing

miRNA from GASC and their respective exosomes, normalized for the amount of producing cells and NTA particle number, were extracted using mirVana PARIS kit (Thermo Fisher, Walthman, MA, USA) following manufacturer’s instructions.

A total amount of 2 μg was analyzed using the “TruSeqSmallRNA Sample Prep kit” (Illumina, San Diego, CA, USA) for library preparation following the manufacturer’s instructions. Both RNA samples and final libraries were quantified by using the Qubit 2.0 Fluorometer (Invitrogen, Carlsbad, CA, USA) and quality tested by Agilent 2100 Bioanalyzer RNA Nano assay (Agilent technologies, Santa Clara, CA, USA). Libraries were then processed with Illumina cBot for cluster generation on the flowcell, following the manufacturer’s instructions, and sequenced on single-end 50 bp mode on HiSeq2500 (Illumina, San Diego, CA, USA), averaging 12 million sequenced reads per sample. The CASAVA 1.8.2 version of the Illumina pipeline was used to process raw data for both format conversion and de-multiplexing. Upon adapter removal with Cutadapt [45], fragments were mapped to the miRbase [46] release 20 and human genome hg19 databases. Cufflinks [47], Cuffdiff [48], and the edgeR R/Bioconductor package [49] were used to evaluate gene expression and pairwise differential expression, respectively.

### 2.6. miRNA Targets Functional Enrichment Analysis

We retrieved differentially expressed miRNA (DE-miRNA) human validated targets through the DIANA-mirPath v.3 web-server [50]. Functional enrichment analysis was performed querying the Kyoto Encyclopedia of Genes and Genomes (KEGG) database (*p* ≤ 0.05), applying the “pathways union” method and retaining the top15 most significantly enriched terms. DIANA-miRPath v3.0 extends the Fisher’s exact test, EASE score, and false discovery rate methodologies, with the use of unbiased empirical distributions.

### 2.7. Survival Analysis

miRNA expression data (HiSeq, miRgene level; RPM, Log2(Val+1); no distinction could be made between the -5p and -3p forms) and clinical data were downloaded from the LinkedOmics portal for TCGA-LGG lower grade glioma patients (*n* = 512 and *n* = 515, respectively; http://linkedomics.org/data_download/TCGA-LGG/). We removed patients that lacked either expression (*n* = 1) or overall survival data (*n* = 34), defining the final cohort (*n* = 477) that was used for the following studies. The prognostic value of the four examined DE-miRNA signatures was evaluated using the RTCGA, clinical and survival R packages. We stratified patients into “high-expression” and “low-expression” based on p-value optimization using the “surv_cutpoint” function (minprop = 0.33). The difference in overall survival rates between the two subgroups was verified applying a log-rank test (*p*-value < 0.05), and Kaplan–Meier plots were finally drawn to summarize the data. We finally performed a multivariate Cox regression analysis to evaluate the prognostic significance of DE-miRNA signatures with respect to clinical covariates (sex, age, and histological subtype).

## 3. Results

### 3.1. Characterization of Exosomes Derived from LGG with Different Prognosis

**Figure 1:**
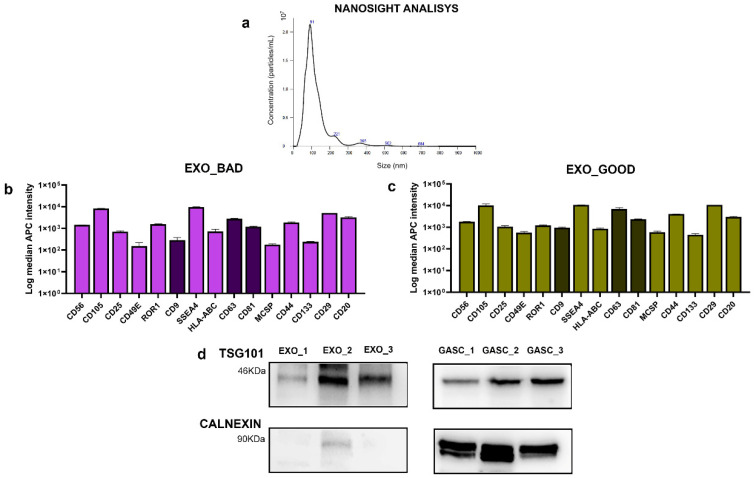
(**a**) Representative size distribution of an EXO_GOOD sample. Surface phenotype of (**b**) EXO_BAD and (**c**) EXO_GOOD exosomes: histograms represent, as mean ± standard deviation, the Log median Allophycocyanin (APC) intensity of 15 markers. (**d**) Representative Western blot for exosome-specific marker (TSG-101) and for cell endoplasmic reticulum marker (calnexin) on exosomes lysates (left) and on paired producing GASC_BAD cells (right).

GASC cells were isolated from *n* = 3 LGG patients who underwent anaplastic transformation within 48 months (GASC_BAD) and *n* = 3 LGG patients who underwent anaplastic transformation after 7 years (GASC_GOOD). Exosomes, defined as EXO_BAD and EXO_GOOD, depending on the GASC of origin, were precipitated from GASC supernatants through polymer-based precipitation and further characterized. Figure 1a shows a representative nanoparticle tracking analysis of an examined sample. Size data support the exosome nature of the particles, being within a range of 50 and 150 nm. Overall, EXO_GOOD and EXO_BAD did not significantly differ in terms of size (91 ± 10 nm vs. 95 ± 10 nm; *p*-value: 0.35) and concentration (1.9 ± 0.3 × 10^12^ particles/mL vs. 1.6 ± 0.2 × 10^12^ particles/mL; *p*-value: 0.52).

To characterize GASC-derived exosomes, we performed MACSPLEX analysis and Western blot. Figure 1b,c show the surface immunophenotype of EXO_BAD and EXO_GOOD. As reported, all nanoparticle preparations expressed, consistently with exosome standards, the tetraspanins CD9, CD81, and CD63. Moreover, all GASC exosomes expressed high levels of the surface markers CD105, SSEA4, CD44, CD29, and CD20 and low levels of CD56, CD25, CD49E, ROR1, HLA-ABC, MCSP, and CD133.

Figure 1d shows Western blots performed on representative exosome preparations for the detection of the TSG101 protein, a key component of the ESCRT-I complex (endosomal sorting complex required for transport), commonly used as protein marker for exosomes, and calnexin, marker protein for the endoplasmic reticulum. As shown, TSG101 is expressed in all exosome preparations, and calnexin, although detectable in exosomes, is strongly depleted compared to GASC secreting cells.

All data suggest that the particles isolated from GASC supernatant, either GASC_GOOD or GASC_BAD, presented size and surface phenotype typical of exosomes.

### 3.2. EXO_GOOD and EXO_BAD Display Different miRNA Profiles

RNAs extracted from GASC_GOOD, EXO_GOOD, GASC_BAD, and EXO_BAD samples were used for miRNA profiling by NGS. A total of 2425 mature miRNAs were efficiently obtained and sequenced. Moreover, we identified *n* = 493 miRNAs with normalized average expression > 20 counts in the profiled samples. Table 1 shows the lists of miRNAs either up- or downregulated in exosomes with respect to their cellular counterparts (i.e., EXO_GOOD vs. GASC_GOOD and EXO_BAD vs. GASC_BAD).

**Figure 2:**
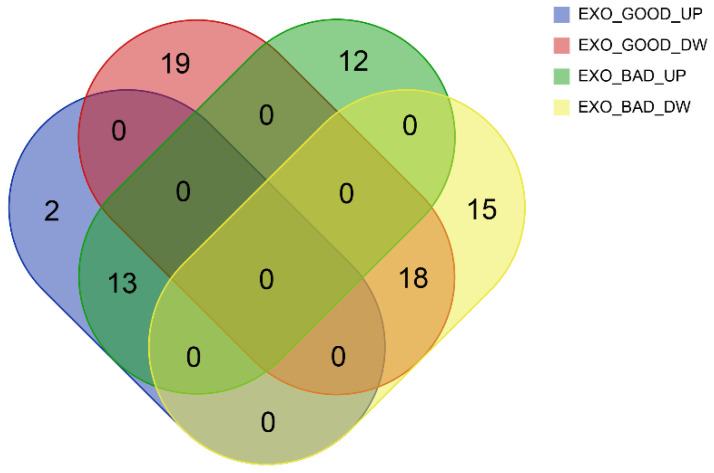
Venn diagram displaying the intersection in miRNAs of EXO_GOOD_UP (blue), EXO_GOOD_DOWN (red), EXO_BAD_UP (green), and EXO_BAD_DOWN (yellow).

As reported in Table 1 and Figure 2, some miRNAs were upregulated (*n* = 13) or downregulated (*n* = 18) in both EXO_GOOD and EXO_BAD, with respect to their cellular counterparts. This suggests the existence of a baseline miRNA profile shared by LGG exosomes, independently from disease aggressiveness.

Considering miRNAs distinctively regulated in EXO_GOOD and EXO_BAD, we observed, in the first group, two miRNAs upregulated, and 19 miRNAs downregulated, while, in the second one, 12 miRNAs upregulated and 15 downregulated.

All lists of miRNAs with logFC, *p*-value, and FDR are displayed in Supplementary Table S1.

### 3.3. Most of Modulated miRNAs in EXO_GOOD and EXO_BAD Are Endowed with Prognostic Significance

Before further investigating the differentially expressed miRNAs, we decided to evaluate the ability of these miRNA signatures to predict overall survival (OS) in 477 newly diagnosed diffuse LGG patients comprised within The Cancer Genome Atlas (TCGA) database.

**Figure 3:**
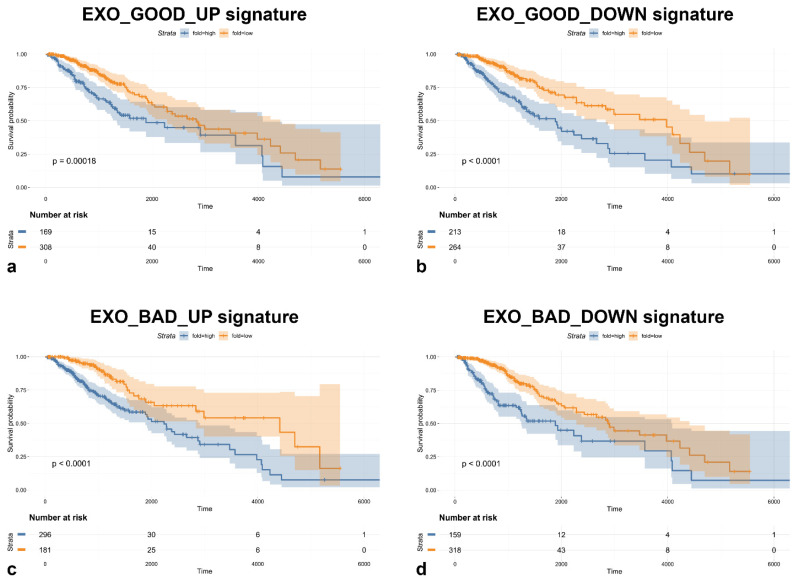
Kaplan–Meier curves showing overall survival (OS) of low-grade gliomas (LGG) patients stratified using (**a**) EXO_GOOD_UP, (**b**) EXO_GOOD_DOWN, (**c**) EXO_BAD_UP, and (**d**) EXO_BAD_DOWN signatures (*p*-value optimization using the ‘surv_cutpoint’ function, minprop = 0.33).

All miRNA signatures identified significantly predicted prognosis, as shown in Figure 3. Moreover, univariate analysis showed the same trend for all the signatures tested, indicating that almost all miRNAs found in LGG GASC exosomes, either with good or bad prognosis, once expressed at high levels, correlated with a poorer overall survival. These data are very interesting, as we were expecting to find an opposite trend for upregulated or downregulated signatures, being the first ones associated with a bad prognosis and the second ones with a good prognosis. The fact that both signatures are, instead, endowed with a poor prognosis suggests that the tumor-supporting function of exosomes is mainly mediated by the transfer of oncogenic miRNAs rather than by the reduced transfer of tumor suppressive miRNAs. miRNAs downregulated in exosomes are possibly the expression of oncogenic miRNAs, which act mainly at the intracellular level.

**Figure 4:**
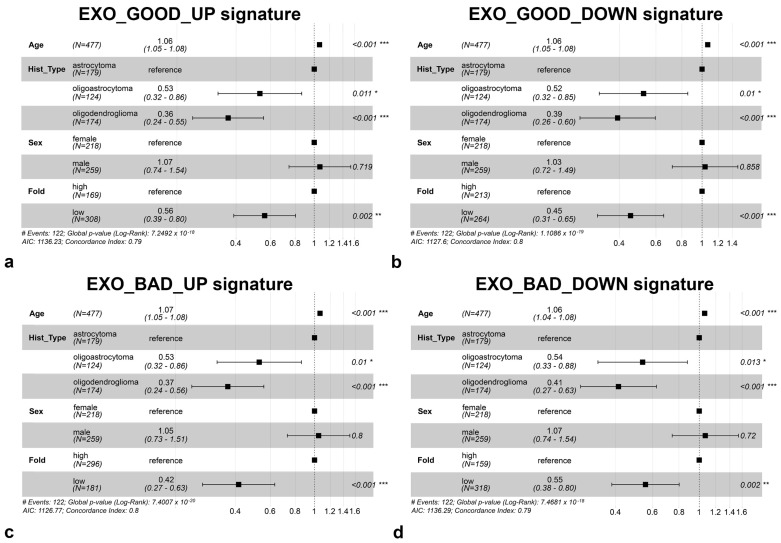
Forest plot displaying multivariate analysis including miRNA signatures of (**a**) EXO_GOOD_UP, (**b**) EXO_GOOD_DOWN, (**c**) EXO_BAD_UP, and (**d**) EXO_BAD_DOWN as well as age, histological subtype, and sex. Significance level: * 0.05 < *p*-value ≤ 0.01; ** 0.01 < *p*-value ≤ 0.001; *** *p*-value < 0.001.

To further validate the prognostic value of these signatures, we performed a multivariate analysis, displayed in Figure 4, including other parameters such as histological subtype, age, and sex. As shown, even though the last parameter did not correlate with prognosis, age and histological subtype were significantly capable to predict prognosis, along with the exosome miRNA signatures.

All data reported suggest that exosomes derived from the TME of LGG have a miRNA content endowed with a significant prognostic value.

### 3.4. Functional Enrichment Analysis Reveals Common Pathways in EXO_GOOD and EXO_BAD

As all miRNA subsets, once expressed at high levels, predicted a poorer OS, we decided to further investigate the possible mechanisms responsible for the worse prognosis of some LGG. For this reason, we proceeded with the functional annotation of the miRNA subsets upregulated and downregulated in EXO_GOOD and EXO_BAD. Table 2 and Table 3 list the 15 most statistically significant KEGG pathways affected by the dysregulated miRNAs in EXO_GOOD_UP, EXO_GOOD_DOWN, EXO_BAD_UP, and EXO_BAD_DOWN subsets along with the number of target genes and miRNAs involved, in each subset, in the different KEGG pathways. Supplementary Table S2 reports all the results obtained using DIANA-mirPATH with all the lists of miRNAs, the list of targets for each miRNA, and the complete KEGG pathway annotation.

As expected, upregulated and downregulated miRNAs acted on different KEGG pathways, while there was a strong similarity between KEGG pathways modulated by EXO_GOOD and EXO_BAD (Table 2 and Table 3). In fact, downregulated miRNAs, independently from the origin, EXO_GOOD or EXO_BAD, were mainly acting on cell metabolism, cell growth, and cell–cell matrix interaction. Upregulated miRNAs were instead modulating KEGG pathways associated with different cancers. In order to understand how EXO_BAD could be responsible for a more aggressive phenotype, we focused our attention on two KEGG pathways, i.e., “Glioma” and “Proteoglycans in cancer”, modulated by both up- and downregulated exosomes, either in EXO_BAD or EXO_GOOD.

### 3.5. Specific miRNAs of EXO_BAD Could Provide Insight into the Poorer Outcome of Some LGG

Since we were interested in understanding how the miRNA content of EXO_BAD could be responsible for the more aggressive phenotype of LGG_BAD, we focused our attention on EXO_BAD miRNAs.

Considering the lists of miRNAs that in EXO_BAD were included in “Glioma” and “Proteoglycans in cancer” KEGG pathways (Supplementary Table S3), we noticed that, while some miRNAs were similarly regulated in EXO_GOOD (i.e., upregulated or downregulated in both EXO_GOOD and EXO_BAD), others were selectively modulated only in EXO_BAD. We hypothesized that the first group of miRNAs could represent a shared signature of LGG exosomes, independent from tumor aggressiveness, while the second one an exosome signature specific for LGG with a bad prognosis. Therefore, among the EXO_BAD miRNAs involved in the KEGG pathways of “Glioma” (Table 4) and “Proteoglycans in cancer” (Table 5), we distinguished miRNAs dysregulated also in EXO_GOOD (i.e., EXO-SHARED miRNAs) and miRNAs selectively modulated in EXO_BAD (i.e., BAD-SPECIFIC miRNAs). All the target genes associated with each up- or downregulated miRNA subsets are reported in Supplementary Table S4.

**Figure 5:**
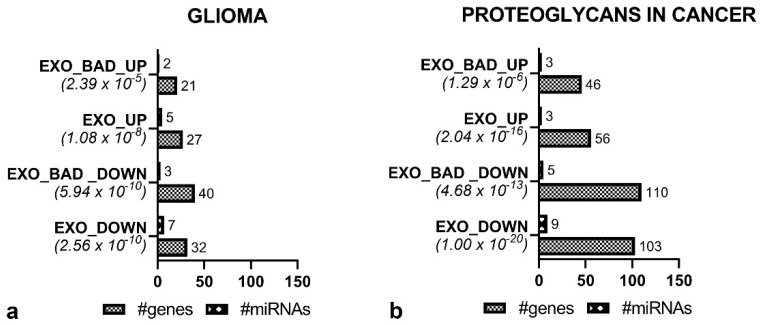
Histograms displaying the *p*-value, number of genes, and number of miRNAs involved in the KEGG pathways (**a**) “Glioma” and (**b**) “Proteoglycans in cancer”, distinguishing upregulated (UP) and downregulated (DOWN) as well as BAD-SPECIFIC (EXO_BAD) and EXO-SHARED (EXO) miRNAs.

Then, considering the KEGG pathways “Glioma” (Figure 5a) and “Proteoglycans in cancer” (Figure 5b), we evaluated how many miRNAs of each group (BAD-SPECIFIC and shared with GOOD) were involved, how many genes of the pathways could be modulated by upregulated or downregulated miRNAs, and the significance of the association.

Focusing on the “Glioma” KEGG pathway, we comprehensively recognized, in EXO_BAD, seven upregulated miRNAs modulating 33 predicted genes and 10 downregulated miRNAs modulating 47 genes (Table 3). Interestingly, the two BAD-SPECIFIC upregulated miRNAs and the three BAD-SPECIFIC downregulated miRNAs were able to modulate, respectively, 65 (21/33) and 85% (40/47) of the predicted target genes (Figure 5a). Similarly, the three BAD-SPECIFIC upregulated and the five BAD-SPECIFIC downregulated miRNAs in EXO_BAD were able to modulate 57.5 and 80.3% of the predicted genes included in the “Proteoglycans in cancer” KEGG pathway, respectively (Table 3 and Figure 5b). As a whole, this suggests that, in spite of their low number, BAD-SPECIFIC miRNAs could regulate most of the predicted target genes included in the two considered KEGG pathways.

**Figure 6:**
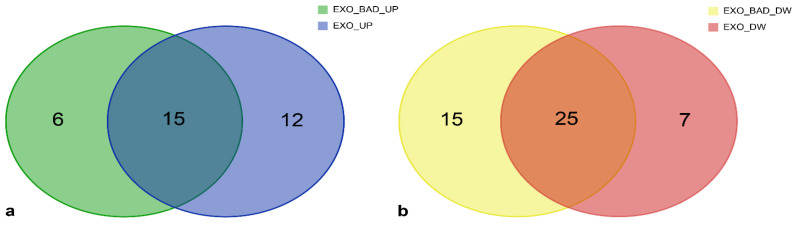
Glioma KEGG pathway: (**a**) Number of validated target genes modulated exclusively by upregulated “BAD-SPECIFIC” (green), “EXO-SHARED” (blue), or by both groups of miRNAs (greenish blue). (**b**) Number of validated target genes modulated exclusively by downregulated “BAD-SPECIFIC” (yellow), “EXO-SHARED” (red), or by both groups of miRNAs (orange). Genes belonging to each group are listed in Table 6.

**Figure 7:**
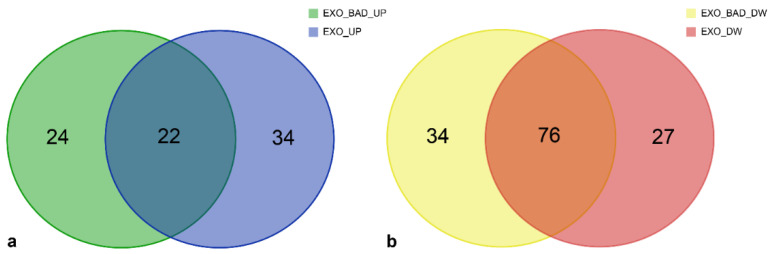
“Proteoglycans in cancer” KEGG Pathway: (**a**) Number of validated target genes modulated exclusively by upregulated “BAD-SPECIFIC” (green), “EXO-SHARED” (blue), or by both groups of miRNAs (greenish blue). (**b**) Number of validated target genes modulated exclusively by downregulated “BAD-SPECIFIC” (yellow), “EXO-SHARED” (red), or by both groups of miRNAs (orange). Genes belonging to each group are listed in Table 7.

We further explored the genes, included in the “Glioma” (Figure 6 and Table 6) and “Proteoglycans in cancer” (Figure 7 and Table 7) KEGG pathways, regulated by miRNAs either BAD-SPECIFIC or EXO-SHARED.

In fact, considering the 33 genes of the “Glioma” pathway modulated by upregulated miRNAs, six were exclusively targeted by BAD-SPECIFIC miRNAs, while the remaining were targets of either EXO-SHARED (*n* = 12) or both EXO-SHARED and BAD-SPECIFIC (*n* = 15) miRNAs (Figure 6). Similarly, 15 out of 47 target genes of downregulated miRNAs were modulated by BAD-SPECIFIC miRNAs. Table 6 lists the validated genes targeted by BAD-SPECIFIC miRNAs, EXO-SHARED miRNAs, or both.

Concerning the “Proteoglycans in cancer” KEGG pathway, as reported in Figure 7 and Table 7, among the 80 modulated genes of upregulated miRNAs, 24 were exclusively targeted by BAD-SPECIFIC miRNAs, while the remaining were targets of either EXO-SHARED (*n* = 34) or both EXO-SHARED and BAD-SPECIFIC (*n* = 22) miRNAs. Similarly, among the 137 genes targeted by downregulated miRNAs, 34 were modulated only by BAD-SPECIFIC miRNAs, while the remaining were targets of either EXO-SHARED (*n* = 27) or both EXO-SHARED and BAD-SPECIFIC (*n* = 76) miRNAs.

Altogether, data suggest that, EXO_BAD and EXO_GOOD can generally act on the same KEGG targets but also specifically on additional elements of the considered pathway.

## 4. Discussion

WHO grade II gliomas, here defined as LGG, represent a group of usually slow-growing primary malignant brain tumors, which, however, in 70% of cases undergo recurrence and/or transformation into tumors with greater malignancy [4]. Thus, LGG outcome is heterogeneous, with an overall survival ranging from 2 to over 12 years. Predicting the outcome and distinguishing low-risk and high-risk patients is fundamental to assist clinicians in the decision-making process aimed at not either overtreating or undertreating patients [3,4,9]. Although age (>40 years), incomplete extent of resection, radiological progression, and/or neurological symptoms contribute to recognizing high-risk patients, a better prognostic stratification is required [3,4,9]. The molecular classification of LGG into three classes (IDH wildtype, IDH mutant only, and IDH mutant and 1p/19q co-deleted), according to WHO criteria [1], has further contributed to recognize patients at risk of quick progression [5,6,7]. Beside prognostic stratification, the choice of adjuvant treatments also remains challenging. Available therapies, radiotherapy and chemotherapy, are burdened by important side effects (e.g., late cognitive defects after radiotherapy), which limit their use in patients with a long life expectancy.

For this reason, there is an increasing interest of the scientific community in the TME [12,13,39,43]. The TME consists of various types of non-tumor cells, such as endothelial cells, stromal cells, pericytes, immune cells, and components of the extracellular matrix. The bidirectional crosstalk between tumor cells and TME contributes to tumor development and progression by promoting tumor cell proliferation, invasion, and angiogenesis; reprogramming of energy metabolism, and suppressing cell death and immune response, well-known hallmarks of cancer [12,13,39,43]. It has been shown that the TME can predict prognosis, and it is considered an interesting therapeutic target [51]. It has been suggested that more than ablation of the TME, it might be useful to interrupt the bidirectional crosstalk with tumor cells or to reverse its phenotype by re-establishing a microenvironment capable of counteracting tumor growth [51]. It has been shown that, in the tumor, both cancer cells and tumor-associated stromal cells promote tumor-induced immune suppression, angiogenesis, and metastasis through the release of exosomes [18,19,20,27]. The latter, through their content in biologically active molecules, including miRNAs, can act as a potent intercellular communication system [18,22,28,52,53].

To better investigate the role of the TME in glioma, we have isolated from low-grade and high-grade gliomas a population of stem cells, named glioma-associated stem cells (GASC). GASC are a population of tumor derived stem cells representative of the TME: although devoid of the genetic alterations of the tumor cells, they are able to support, in vitro, the tumor growth [41]. Additionally, we demonstrated that GASC derived from LGG can independently predict patient prognosis [41], and, from the transcriptomic analysis of GASC derived from LGG with different prognosis, it was possible to identify a prognostic gene signature further validated in 530 newly diagnosed diffuse LGG patients comprised within The Cancer Genome Atlas (TCGA) database [42]. Importantly, GASC exert their tumor-supporting function through the release of exosomes [41,44]. Specifically, GASC-derived exosomes are able to increase proliferation, motility, and anchorage-independent growth of both commercially available glioblastoma cell lines and patient-derived glioma stem cells (GSC) [41,44].

To obtain insights into the TME-related mechanisms, possibly responsible of the more aggressive behavior of some LGG, we isolated exosomes from the supernatant of GASC derived from both LGG patients undergoing a rapid malignant transformation (EXO_BAD) and LGG patients experiencing malignant transformation after at least 7 years (EXO_GOOD).

EXO_BAD and EXO_GOOD were produced with the same yield from GASC_GOOD and GASC_BAD and shared similar size, concentration, and markers that ascribed them as exosomes. However, some differences could be detected in their miRNA content. It is important to notice that, although ultrafiltration using 100 kDa molecular weight cutoffs could remove some RNPs, we cannot exclude the presence of ribonucleoprotein particles complexes or high-density lipoproteins. Still, the samples were isolated in the same conditions resulting in a shared medium-derived contaminant background throughout the experiment.

By taking advantage of deep sequencing technologies, we analyzed the miRNA profiles of both exosomes and their producing cells. With respect to their cellular counterpart, exosomes were either enriched in some miRNAs or depleted in others. This is consistent with what previously published: as shown for mRNA [28,54,55,56], miRNAs also seem not to be randomly selected and loaded into exosomes [57,58,59].

Comparing the miRNAs enriched or depleted in EXO_GOOD and EXO_BAD, with respect to their cellular counterparts, we identified: 1. a large group of miRNAs similarly regulated in the two groups and 2. miRNAs differently regulated in EXO_GOOD and EXO_BAD.

Focusing our attention on the miRNA signature shared by GASC exosomes, independently from patient prognosis, we observed either upregulation or down regulation of both oncosuppressive and oncogenic miRNAs. For example, with respect to their cellular counterpart, GASC exosomes resulted in the depletion of many members of the let-7 family. This latter exerts a tumor suppressive function by inhibiting proliferation and malignancy of glioblastoma cells by targeting both NRAS and KRAS [60,61] and by modulating microglia activation through TLR7 [62]. On the other hand, GASC exosomes resulted in the depletion of miR-21 and miR-221, two miRNAs whose oncogenic role in glioblastomas has been widely demonstrated [63,64,65,66]. Similarly, GASC exosomes were enriched in miR-451 and miR-150, which are known to inhibit GSC growth [67] and glioma cell proliferation, invasion and apoptosis through the Akt pathway [68] and to suppress glioma cell proliferation and migration by targeting MMP1, respectively [69]. Conversely, GASC exosomes were enriched in miR-1246, responsible for glioma immune escape by targeting TERF2IP [70], and in miR-4516, known to exert a tumor promoting function by regulating the Hippo pathway in glioblastoma [71]. It would be extremely interesting to compare this upregulated and downregulated “baseline” miRNA signature with non-transformed glial cells, to assess if this signature could be representative of glial cells.

Looking instead to the two miRNAs specifically enriched in EXO_GOOD, miR-320c has been shown to exert a tumor suppressive function in glioma, by acting through the modulation of either RAF1/MAPK pathway or E2F2, a key transcription factor involved in the regulation of the G1/S phase [72,73,74]. Li et al. reported that miR-320c could increase radiosensitivity of glioma cell by inhibiting SIRT1 through FOXM1 modulation [74]. No information is instead available on a possible role of miR-4532 in glioma. Regarding the miRNAs selectively upregulated in GASC_BAD, an oncogenic role, in glioma, has been described for miR-223-3p [75], miR-10b-5p [76,77], and miR-182-5p [78,79], while miR-338-3p [80,81], miR-204-5p [82], and miR-126-3p [83] were described as oncosuppressive. Instead, few or no information on the role of miR-4492 [84], miR-4508, miR-7704, miR-4488 [85], and miR-151b on glioma are available.

To better understand these data of non-univocal interpretation, we decided to verify the possible clinical significance of the identified exosome signatures in 477 newly diagnosed diffuse LGG patients comprised within the TCGA database [42]. We considered two signatures corresponding to the miRNA upregulated in EXO_GOOD and in EXO_BAD and two signatures corresponding to the miRNA downregulated in EXO_GOOD and in EXO_BAD. Interestingly, all four signatures, if upregulated, had negative prognostic significance. This suggests that miRNA signatures, although composed of oncogenic and oncosuppressive miRNAs, are globally the representation of a more aggressive tumor phenotype. Additionally, the fact that both upregulated and downregulated miRNAs were associated with a poor prognosis suggests that the tumor supportive function of exosomes is likely mediated by the delivery of over-expressed miRNAs rather than by the reduced transfer of tumor suppressor miRNAs. In fact, even miRNAs whose expression is reduced in exosomes were oncogenic. We can therefore hypothesize that they represent a class of oncogenic miRNA mainly retained at intracellular level.

Consistently, when we proceeded with the functional annotation of miRNAs upregulated and downregulated in EXO_GOOD and EXO_BAD, we noticed that, independently from the GASC of origin, upregulated and downregulated miRNAs were targeting different KEGG pathways. In fact, downregulated miRNAs, either in EXO_GOOD or EXO_BAD, were mainly acting on cell metabolism, cell growth, and cell–cell matrix interaction. This supports the hypothesis that an intracellular enrichment of these miRNAs could confer a more aggressive tumor phenotype. Upregulated miRNAs were instead modulating KEGG pathways associated with different cancers, including glioma, chronic myeloid leukemia, prostate carcinoma, non-small cell carcinoma, renal cell carcinoma, and pancreatic carcinoma. This suggests that exosomes can transfer miRNAs able to modulate a more general “tumor-related mechanism” globally shared by different cancers.

As mentioned, when we compared the KEGG pathways modulated by upregulated EXO_BAD and EXO_GOOD, no major differences were detected. Same observation could be done for downregulated miRNAs in EXO_GOOD and EXO_BAD.

Therefore, to get insights into the mechanisms by which EXO_BAD could exert a stronger tumor-supporting function, we focused our attention on two KEGG pathways shared by all the four exosome-related signatures: “Glioma” and “Proteoglycan in cancer”. The first one was selected because it strictly related to our tumor setting. The second one because it is known that proteoglycans can regulate cell signaling and migration, as well as immune response, by interacting with both extracellular (extracellular ligands, growth factor receptors, and extracellular matrix) and intracellular (enzymes and structural proteins) elements [86]. Therefore, proteoglycans are key players in intracellular oncogenic pathways as well as in tumor–TME crosstalk [86]. Additionally, in glioma, it has been demonstrated that the expression of heparan sulfate proteoglycans is significantly increased in high grade gliomas, with respect to LGG [87], and a similar pattern has been demonstrated for the dermatan sulfate proteoglycan endocan [88] and the cell surface chondroitin sulfate proteoglycan CD44 [89].

Restricting our analysis to EXO_BAD, we assessed that, while some miRNAs were similarly upregulated or downregulated in EXO_GOOD, few others were specific of EXO_BAD. We named the first ones EXO-SHARED miRNAs and the latter BAD-SPECIFIC miRNAs. In particular, miR-126-3p and miR-182-5p were BAD-SPECIFIC miRNAs upregulated in both “Glioma” and “Proteoglycans in cancer” KEGG pathways, while miR-223-3p was exclusively upregulated in the second one. Concurrently, three BAD-SPECIFIC miRNAs (miR-29b-3p, miR-34a-5p, and miR-497-5p) were downregulated in both “Glioma” and “Proteoglycans in cancer” pathways, and one, miR-582-3p, exclusively in the “Proteoglycans in cancer” pathway.

miR-182-5p is a well-described oncomiR that, in HGG, is known to promote the proliferative and invasive capacities of tumor cells through the modulation of either STAT3 or NF-κB signaling pathways [78,79,90]. Interestingly, analyzing the target genes comprised in the KEGG pathways, miR-182-5p modulates all the validated targets present in the “Glioma” pathway and most of the targets present in the “Proteoglycans in cancer” pathway. miR-223 is another well-recognized oncogenic miRNA, which, in glioblastoma, promotes growth and invasion of tumor cells by targeting the tumor suppressor PAX6, exerting its function by inhibiting the expression of metalloproteases MMP2 and MMP9 [91]. As mentioned above, the role of miR-126-3p is more controversial, since it has been recognized as an oncosuppressive miRNA [83]. However, more recently, its role in retinal angiogenesis has been demonstrated, both in vitro and in vivo, by protecting endothelial cells from apoptosis [92].

Concerning miRNAs downregulated in the EXO_BAD, miR-34a-5p along with miR-497-5p were the most involved in the modulation of all predicted targets in both “Glioma” and “Proteoglycans in cancer” pathways. miR-34a is a p53 transcriptional target poorly expressed in HGG compared to normal brain tissues [93], and it is known to suppress in vitro and in vivo tumor growth by targeting c-MET and NOTCH, thus directly modulating glioma cell cycle [94]. Moreover, miR34a has been reported to modulate glioma cells apoptosis by targeting BCL2 [95] and stimulating senescence in glioma cells by inducing DNA damage [96]. On the other hand, the clinical significance of miR-497 has not been fully elucidated yet, but studies described a significant decrease in this miRNA in HGG with respect to LGG and demonstrated its role in suppressing angiogenesis, by targeting VEGFA [97] and glioma proliferation and epithelial-to-mesenchymal transition, by modulating WNT3A [98]. Moreover, decreased expression of miR-497 was associated, in glioma, with a worse prognosis [97,98].

Therefore, our study suggests that targets specific for the aggressive phenotype in LGG are modulated by miR-182-5p, miR-223-3p, miR-34a-5p, and miR-497-5p. It could be extremely interesting to further investigate these four miRNAs and the role they play in the evolution of LGG.

It is also worth noting that most of the gene targets in the “Glioma” pathway are included in the “Proteoglycans in cancer” pathway, thus confirming the importance of proteoglycans in glioma progression [86,87,88,89].

Finally, considering the genes targeted by either BAD-SPECIFIC or EXO-SHARED miRNAs for each KEGG pathway analyzed, three were the main observations. First, BAD-SPECIFIC miRNAs, even when fewer than the EXO-SHARED ones, were able to modulate most of the target genes included in the considered KEGG pathway. Second, BAD-SPECIFIC miRNAs and EXO-SHARED miRNA presented a certain number of common gene targets, suggesting that there was an increased modulation of these genes in cells capable of internalizing EXO_BAD. Third, BAD-SPECIFIC miRNAs could hit an additional number of gene targets, compared to EXO-SHARED miRNAs. This leads us to hypothesize that the greater tumor-supporting function of EXO_BADs could be linked to their increased ability to modulate target pathways. Several issues remain to be elucidated. The presence of an increased expression or downregulation of both oncosuppressive and oncogenic miRNAs can be due to the fact that LGG are a low-grade tumor anyway, and some miRNAs can be the manifestation of a baseline tumor suppressive signal, which could explain the slower malignant evolution of this neoplasm.

The confirmation that miR-182-5p, miR-223-5p, miR-34a-5p, and miR-497-5p could be putative miRNAs involved in the aggressive phenotype of the tumor requires further in vitro and in vivo study.

Additionally, the mechanisms responsible for the selection of the miRNA cargo remain an interesting field of study; understanding them could get insights into the biology of cancer and the possibility to hijack this intracellular communication system to deliver tumor-suppressive messages able to revert the tumor-supporting function of the TME [51,99]

In conclusion, the widely variable LGG prognosis and the lack of specific adjuvant treatment requires the identification of novel prognostic markers and therapeutic targets. In this regard, our work suggested that patient-derived GASC, stem cells representative of the tumor microenvironment, and their released exosomes could give important insights. We have demonstrated that exosomes released by GASC isolated from LGG characterized by different prognosis possess a miRNA content that can modulate key pathways in tumor progression and aggressiveness. We could identify miRNA signatures endowed with clinical significance, being able to predict prognosis in 477 LGG patients. Additionally, exosomes derived from GASC of patients with a bad prognosis possess a unique repertoire of BAD-SPECIFIC miRNAs, possibly responsible for the more aggressive function. Further studies are required to demonstrate whether this information could lead to novel therapies aimed at targeting the crosstalk between tumor cells and their tumor-supporting microenvironment.

## Figures and Tables

**Table 1:** List of upregulated and downregulated miRNAs in EXO_GOOD, with respect to GASC_GOOD, and EXO_BAD, in comparison with GASC_BAD. miRNAs reported in bold are specific for each subset, while the others are similarly expressed in both EXO_GOOD and EXO_BAD.

	UPREGULATED miRNAs	DOWNREGULATED miRNAs
**EXO_GOOD**	**hsa-miR-320c, hsa-miR-4532**, hsa-miR-122-5p, hsa-miR-1246, hsa-miR-126-5p, hsa-miR-142-3p, hsa-miR-142-5p, hsa-miR-144-3p, hsa-miR-144-5p, hsa-miR-150-5p, hsa-miR-378c, hsa-miR-378d, hsa-miR-4516, hsa-miR-451a, hsa-miR-486-5p	**hsa-let-7d-5p, hsa-let-7g-5p, hsa-miR-100-3p, hsa-miR-1185-1-3p, hsa-miR-125b-5p, hsa-miR-143-5p, hsa-miR-145-3p, hsa-miR-145-5p, hsa-miR-148b-3p, hsa-miR-22-3p, hsa-miR-24-2-5p, hsa-miR-2682-5p, hsa-miR-374a-5p, hsa-miR-377-3p, hsa-miR-381-3p, hsa-miR-424-5p, hsa-miR-487a-3p, hsa-miR-539-3p, hsa-miR-98-5p**, hsa-let-7a-5p, hsa-let-7e-5p, hsa-let-7f-5p, hsa-miR-140-5p, hsa-miR-152-5p, hsa-miR-193a-3p, hsa-miR-193a-5p, hsa-miR-21-3p, hsa-miR-221-5p, hsa-miR-23a-5p, hsa-miR-29a-3p, hsa-miR-337-3p, hsa-miR-34c-5p, hsa-miR-374a-3p, hsa-miR-411-3p, hsa-miR-454-3p, hsa-miR-455-3p, hsa-miR-7974
**EXO_BAD**	**hsa-miR-223-3p, hsa-miR-223-5p, hsa-miR-338-3p, hsa-miR-4492, hsa-miR-4508, hsa-miR-7704, hsa-miR-4488, hsa-miR-10b-5p, hsa-miR-204-5p, hsa-miR-151b, hsa-miR-182-5p, hsa-miR-126-3p**, hsa-miR-122-5p, hsa-miR-1246, hsa-miR-126-5p, hsa-miR-142-5p, hsa-miR-144-3p, hsa-miR-144-5p, hsa-miR-150-5p, hsa-miR-378c, hsa-miR-378d, hsa-miR-4516, hsa-miR-451a, hsa-miR-486-5p	**hsa-miR-335-3p, hsa-miR-497-5p, hsa-miR-29c-3p, hsa-miR-34a-5p, hsa-let-7a-3p, hsa-miR-582-3p, hsa-miR-29b-3p, hsa-miR-137, hsa-miR-378a-5p, hsa-miR-539-5p, hsa-miR-3607-3p, hsa-miR-29b-1-5p, hsa-miR-31-3p, hsa-miR-1304-3p, hsa-miR-190a-5p**, hsa-let-7a-5p, hsa-let-7e-5p, hsa-let-7f-5p, hsa-miR-140-5p, hsa-miR-152-5p, hsa-miR-193a-3p, hsa-miR-193a-5p, hsa-miR-21-3p, hsa-miR-221-5p, hsa-miR-23a-5p, hsa-miR-29a-3p, hsa-miR-337-3p, hsa-miR-34c-5p, hsa-miR-374a-3p, hsa-miR-411-3p, hsa-miR-454-3p, hsa-miR-455-3p, hsa-miR-7974

**Table 2:** Top 15 significant Kyoto Encyclopedia of Genes and Genomes (KEGG) pathways associated with the specific lists of miRNAs dysregulated in EXO_GOOD_UP and EXO_GOOD_DOWN, respectively. Table reports the *p*-value, number of genes, and miRNAs involved in the annotated KEGG pathways.

EXO_GOOD_UP				EXO_GOOD_DOWN			
KEGG Pathway	*p*-Value	#Genes	#miRNAs	KEGG Pathway	*p*-Value	#Genes	#miRNAs
**Proteoglycans in cancer**	1.50 × 10^−9^	56	3	Prion diseases	0	11	4
Viral carcinogenesis	8.92 × 10^−9^	59	6	Fatty acid biosynthesis	0	5	8
**Glioma**	2.52 × 10^−6^	30	6	Steroid biosynthesis	0	9	9
Chronic myeloid leukemia	5.74 × 10^−6^	34	4	Fatty acid metabolism	0	19	9
MicroRNAs in cancer	1.56 × 10^−4^	26	4	Viral carcinogenesis	0	119	11
Non-small cell lung cancer	3.37 × 10^−4^	21	2	Hippo signaling pathway	0	91	15
Renal cell carcinoma	4.01 × 10^−3^	23	3	ECM-receptor interaction	0	39	17
Prostate cancer	4.31 × 10^−3^	33	3	**Proteoglycans in cancer**	0	132	17
Adherens junction	4.44 × 10^−3^	19	4	Cell cycle	3.33 × 10^−16^	79	10
Central carbon metabolism in cancer	4.74 × 10^−3^	19	3	Adherens junction	8.88 × 10^−16^	58	14
Other types of O-glycan biosynthesis	5.85 × 10^−3^	9	2	Lysine degradation	2.89 × 10^−15^	26	11
Pancreatic cancer	7.21 × 10^−3^	27	3	Pathways in cancer	1.69 × 10^−13^	197	12
Lysine degradation	9.84 × 10^−3^	12	3	Hepatitis B	8.91 × 10^−12^	77	9
TGF-beta signaling pathway	1.04 × 10^−2^	25	3	**Glioma**	1.70 × 10^−10^	43	14
FoxO signaling pathway	1.10 × 10^−2^	38	2	TGF-beta signaling pathway	1.13 × 10^−9^	56	12

**Table 3:** Top 15 significant KEGG pathways associated with the specific lists of miRNAs dysregulated in EXO_BAD_UP and EXO_BAD_DOWN, respectively. Table reports the *p*-value, number of genes, and miRNAs involved in the annotated KEGG pathways.

EXO_BAD_UP				EXO_BAD_DOWN			
KEGG Pathway	*p*-Value	#Genes	#miRNAs	KEGG Pathway	*p*-Value	#Genes	#miRNAs
Fatty acid biosynthesis	0	3	3	Prion diseases	0	7	2
Viral carcinogenesis	2.22 × 10^−16^	87	9	Fatty acid biosynthesis	0	4	8
**Proteoglycans in cancer**	2.72 × 10^−11^	80	6	Fatty acid metabolism	0	22	9
Prion diseases	1.08 × 10^−10^	1	1	Viral carcinogenesis	0	121	10
Chronic myeloid leukemia	1.00 × 10^−8^	48	7	Hippo signaling pathway	0	83	14
**Glioma**	6.75 × 10^−6^	33	7	**Proteoglycans in cancer**	0	137	14
Adherens junction	1.59 × 10^−5^	29	5	ECM-receptor interaction	0	43	19
Non-small cell lung cancer	3.00 × 10^−5^	31	5	Steroid biosynthesis	1.11 × 10^−16^	6	9
Prostate cancer	2.55 × 10^−4^	45	5	Adherens junction	7.77 × 10^−16^	57	12
Pancreatic cancer	4.83 × 10^−4^	38	5	Lysine degradation	2.00 × 10^−15^	23	9
Renal cell carcinoma	2.50 × 10^−3^	32	5	p53 signaling pathway	1.12 × 10^−11^	51	10
Other types of O-glycan biosynthesis	2.70 × 10^−3^	11	3	**Glioma**	2.96 × 10^−11^	47	10
Hepatitis B	3.16 × 10^−3^	47	4	Cell cycle	1.02 × 10^−10^	85	6
Pathways in cancer	3.39 × 10^−3^	125	4	Hepatitis B	9.02 × 10^−10^	90	8
Endometrial cancer	8.10 × 10^−3^	21	4	Pathways in cancer	9.96 × 10^−9^	225	9

**Table 4:** List of downregulated and upregulated miRNAs in EXO_BAD involved in the KEGG pathway of “Glioma”, distinguishing miRNAs selectively modulated in EXO_BAD (BAD-SPECIFIC) from those similarly modulated in EXO_GOOD (EXO-SHARED).

GLIOMA	
**UPREGULATED**	**BAD-SPECIFIC: hsa-miR-126-3p, hsa-miR-182-5p** **EXO-SHARED:** hsa-miR-122-5p, hsa-miR-1246, hsa-miR-126-5p, hsa-miR-142-5p, hsa-miR-378d
**DOWNREGULATED**	**BAD-SPECIFIC: hsa-miR-29b-3p, hsa-miR-34a-5p, hsa-miR-497-5p** **EXO-SHARED:** hsa-let-7a-5p, hsa-let-7e-5p, hsa-let-7f-5p, hsa-miR-193a-3p, hsa-miR-29a-3p, hsa-miR-34c-5p, hsa-miR-454-3p

**Table 5:** List of downregulated and upregulated miRNAs in EXO_BAD involved in the KEGG pathway of “Proteoglycans in cancer”, distinguishing miRNA selectively modulated only in EXO_BAD (BAD-SPECIFIC) from those similarly modulated in EXO_GOOD (EXO-SHARED).

PROTEOGLYCANS IN CANCER	
**UPREGULATED**	**BAD-SPECIFIC: hsa-miR-126-3p, hsa-miR-182-5p, hsa-miR-223-3p** **EXO-SHARED:** hsa-miR-122-5p, hsa-miR-126-5p, hsa-miR-142-3p
**DOWNREGULATED**	**BAD-SPECIFIC: hsa-miR-29b-3p, hsa-miR-29c-3p, hsa-miR-34a-5p, hsa-miR-497-5p, hsa-miR-582-3p** **EXO-SHARED:** hsa-let-7a-5p, hsa-let-7e-5p, hsa-let-7f-5p, hsa-miR-140-5p, hsa-miR-193a-3p, hsa-miR-21-3p, hsa-miR-29a-3p, hsa-miR-374a-3p, hsa-miR-454-3p

**Table 6:** “Glioma” KEGG pathway: list of validated target genes modulated only by “BAD-SPECIFIC”, “EXO-SHARED”, or by both “BAD-SPECIFIC” and “EXO-SHARED” miRNAs (COMMON TARGETS).

miRNAs	VALIDATED TARGETS	
**UPREGULATED**	**“BAD-SPECIFIC” only**	*AKT3, CDKN1A, NRAS, PIK3R1, RB1, SHC1*
	**COMMON TARGETS**	*AKT1, CALM1, CCND1, CDK4, CDK6, E2F1, E2F3, IGF1R, MAPK1, PIK3CA, PIK3CB, PIK3CD, PIK3R2, SOS1, TP53*
	**“EXO-SHARED” only**	*CALM2, E2F2, EGFR, GRB2, KRAS, MDM2, MTOR, PIK3CG, PLCG1, PTEN, RAF1, SOS2*
**DOWNREGULATED**	**“BAD-SPECIFIC” only**	*AKT1, AKT3, ARAF, CDKN2A, E2F2, GRB2, IGF1, MAP2K1, MAP2K2, MTOR, PIK3CA, PRKCB, RAF1, SOS2, TGFA*
	**COMMON TARGETS**	*AKT2, BRAF, CALM1, CALM2, CALM3, CCND1, CDK4, CDK6, CDKN1A, E2F1, E2F3, EGFR, IGF1R, MAPK1, MDM2, NRAS, PDGFRA, PDGFRB, PIK3R1, PIK3R2, PIK3R3, PLCG1, PTEN, SOS1, TP53*
	**“EXO-SHARED” only**	*CAMK2D, KRAS, PDGFA, PDGFB, PIK3CB, PRKCA, RB1*

**Table 7:** “Proteoglycans in cancer” KEGG pathway: list of validated target genes modulated only by “BAD-SPECIFIC”, “EXO-SHARED”, or by both “BAD-SPECIFIC” and “EXO-SHARED” miRNAs (COMMON TARGETS).

miRNAs	VALIDATED TARGETS	
**UPREGULATED**	**“BAD-SPECIFIC” only**	*AKT3, CDC42, CDKN1A, EIF4B, ELK1, ESR1, FGF2, FZD6, HIF1A, ITPR3, MET, MMP2, MMP9, MSN, NRAS, PIK3CB, PIK3R1, PPP1R1, PRKACB, RPS6KB2, SDC4, SOS1, STAT3, THBS1*
	**COMMON TARGETS**	*AKT1, CBL, CCND1, CTNNB1, CTTN, FRS2, FZD3, IGF1R, IQGAP1, ITGB1, MAPK1, MTOR, PDPK1, PIK3CA, PIK3CD, PIK3R2, RAC1, RDX, ROCK2, SDC2, TP53, VEGFA*
	**“EXO-SHARED” only**	*ACTB, AKT2, ANK1, ARHGEF12, CD44, DCN, EGFR, FGFR1, FLNB, FZD1, FZD4, FZD7, GAB1, GRB2, HCLS1, ITGAV, KRAS, MAPK13, MDM2, MYC, PAK1, PIK3CG, PIK3R5, PLCG1, PTCH1, PTPN11, RAF1, RPS6KB1, SLC9A1, SMAD2, SOS2, VAV2, VMP1, WNT5A*
**DOWNREGULATED**	**“BAD-SPECIFIC” only**	*AKT1, AKT2, ANK3, ARAF, CBLC, CD44, CD63, COL21A1, CTTN, ELK1, FGF2, FZD1, GAB1, GPC1, GRB2, HGF, HPSE, IGF1, ITGA5, ITPR1, MAP2K1, MAP2K2, MAPK12, MMP9, PIK3CA, PPP1R12B, PRKACA, PRKACB, PRKCB, PRKX, PTCH1, RAF1, ROCK2, TFAP4*
	**COMMON TARGETS**	*ACTB, ACTG1, AKT3, ARHGEF12, BRAF, CAV1, CAV2, CCND1, CDC42, CDKN1A, CTNNB1, DDX5, EGFR, EZR, FGFR1, FLNB, FN1, FRS2, FZD5, FZD6, GPC3, HIF1A, HSPG2, IGF1R, IGF2, IQGAP1, ITGAV, ITGB1, ITGB5, ITPR3, MAPK1, MAPK13, MDM2, MET, MMP2, MSN, MTOR, MYC, NRAS, NUDT16L1, PDCD4, PIK3R1, PIK3R2, PIK3R3, PLAU, PLAUR, PLCG1, PPP1CA, PPP1CB, PPP1CC, PPP1R12A, PPP1R12C, PRKCA, PTPN11, PXN, RDX, RPS6, RRAS, SDC1, SDC2, SDC4, SLC9A1, SMAD2, SMO, SOS1, SOS2, SRC, STAT3, TGFB1, TGFB2, THBS1, TP53, VAV2, VEGFA, WNT5A, WNT9A*
	**“EXO-SHARED” only**	*ARHGEF1, CAMK2D, CASP3, CBL, DROSHA, EIF4B, ESR1, FAS, FLNA, FLNC, FZD2, FZD3, FZD4, HOXD10, ITGA2, ITPR2, KDR, KRAS, PIK3CB, PTK2, PTPN6, RAC1, RPS6KB2, TLR4, VMP1, WNT3A, WNT5B*

## Supplementary Materials

The following are available online at https://www.mdpi.com/2227-9059/8/12/564/s1, Table S1: miRNA’s expression in GASC exosomes compared to their cellular counterparts; Table S2: Functional annotation of each miRNA subset (miRNA targets and KEGG pathways); Table S3: miRNA’s subsets involved in the “Glioma” and “Proteoglycans in cancer” KEGG pathways; Table S4: miRNA’s targets involved in the “Glioma” and “Proteoglycans in cancer” KEGG pathways.
